# **Prediction of a clinically effective dose of THY1773, a novel V**_**1B**_ **receptor antagonist, based on preclinical data**

**DOI:** 10.1002/bdd.2273

**Published:** 2021-04-03

**Authors:** Shoko Inatani, Akiko Mizuno‐Yasuhira, Makoto Kamiya, Izumi Nishino, Helene D. Sabia, Hiromi Endo

**Affiliations:** ^1^ Drug Metabolism and Pharmacokinetics Drug Safety and Pharmacokinetics Laboratories Research Headquarters Taisho Pharmaceutical Co., Ltd. Saitama Japan; ^2^ Development Headquarters Taisho Pharmaceutical Co., Ltd. Tokyo Japan; ^3^ Drug Development Taisho Pharmaceutical R&D Inc. NJ USA; ^4^ Clinical Operations Taisho Pharmaceutical R&D Inc. NJ USA

**Keywords:** effective dose prediction, receptor occupancy, THY1773, TS‐121, V_1B_ receptor

## Abstract

THY1773 is a novel arginine vasopressin 1B (V_1B_) receptor antagonist that is under development as an oral drug for the treatment of major depressive disorder (MDD). Here we report our strategy to predict a clinically effective dose of THY1773 for MDD in the preclinical stage, and discuss the important insights gained by retrospective analysis of prediction accuracy. To predict human pharmacokinetic (PK) parameters, several extrapolation methods from animal or in vitro data to humans were investigated. The f_u_ correction intercept method and two‐species‐based allometry were used to extrapolate clearance from rats and dogs to humans. The physiologically based pharmacokinetics (PBPK)/receptor occupancy (RO) model was developed by linking free plasma concentration with pituitary V_1B_ RO by the E_max_ model. As a result, the predicted clinically effective dose of THY1773 associated with 50% V_1B_ RO was low enough (10 mg/day, or at maximum 110 mg/day) to warrant entering phase 1 clinical trials. In the phase 1 single ascending dose study, TS‐121 capsule (active ingredient: THY1773) showed favorable PKs for THY1773 as expected, and in the separately conducted phase 1 RO study using positron emission tomography, the observed pituitary V_1B_ RO was comparable to our prediction. Retrospective analysis of the prediction accuracy suggested that the prediction methods considering plasma protein binding, and avoiding having to apply unknown scaling factors obtained in animals to humans, would lead to better prediction. Selecting mechanism‐based methods with reasonable assumptions would be critical for the successful prediction of a clinically effective dose in the preclinical stage of drug development.

## Graphical abstract

We evaluated preclinical pharmacokinetics of THY1773, a novel arginine vasopressin 1B (V_1B_) receptor antagonist, and predicted its clinically effective dose for major depressive disorder. Retrospective analysis of prediction accuracy demonstrated that selecting mechanism based methods with reasonable assumptions would be critical for the successful prediction of a clinically effective dose in the preclinical stage of drug development.

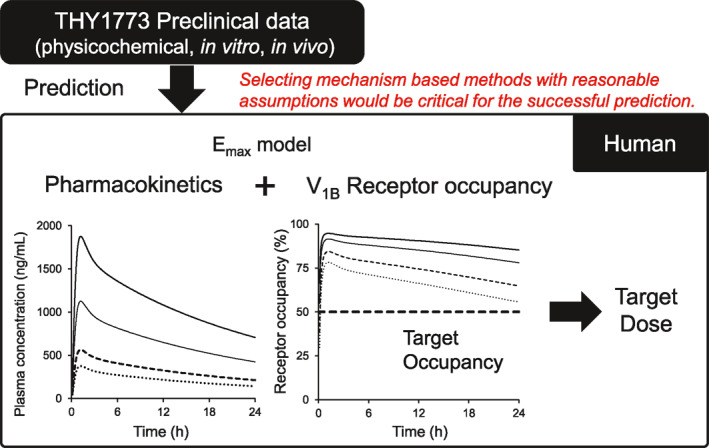

## 1 INTRODUCTION

Dysfunction of the hypothalamic–pituitary–adrenal (HPA) axis activity is observed in a subset of patients with major depressive disorder (MDD) (Holsboer, Haack, Gerken, & Vecsei, 1984). Arginine vasopressin (AVP) plays a crucial role in the regulation of the HPA axis in cooperation with corticotropin‐releasing factor (CRF), and excess AVP is hypothesized to be responsible for HPA axis dysfunction in depression (de Winter et al., 2003; Purba, Hoogendijk, Hofman, & Swaab, 1996). AVP regulates HPA axis activity through adrenocorticotropic hormone (ACTH) secretion from the anterior pituitary by binding to its receptor subtype vasopressin 1B (V_1B_) receptor (Saito, Sugimoto, Tahara, & Kawashima, 1995; Wersinger, Ginns, O’Carroll, Lolait, & Young, 2002). Therefore, the V_1B_ receptor antagonist is expected to ameliorate the abnormalities of the HPA axis observed in depression, and consequently, to improve depressive symptoms in certain subpopulations of depressed individuals with impaired HPA axis function.

To date, several V_1B_ receptor antagonists have been proven to exhibit antidepressant‐like effects in rodent models (Geneste et al., 2018; Griebel et al., 2002; Iijima et al., 2014). Among them, SSR149415, the first orally active V_1B_ receptor antagonist, was tested in double‐blind, placebo‐controlled studies for patients with MDD. However, SSR149415 failed to demonstrate clear antidepressant effects in the trials (Griebel, Beeské, & Stahl, 2012). Although the reason for the failure was not fully understood, it is speculated that dose levels of SSR149415 were insufficient to show efficacy (Griebel , Beeské, & Stahl, 2012). Indeed, in a small scale depression trial (Katz, Locke, Greco, Liu, & Tracy, 2017), another V_1B_ receptor antagonist, ABT‐436, has been reported to be associated with more favorable symptom changes at a dose that inhibits HPA function. Therefore, the therapeutic potential of V_1B_ receptor antagonists for MDD needs to be further investigated, especially at doses that would show adequate blockade of the V_1B_ receptor.

THY1773 is a potent and selective V_1B_ receptor antagonist, which blocks the anterior pituitary V_1B_ receptor and exhibits antidepressant‐like effects in rodents (Kamiya et al., 2020). In rats, THY1773 occupied the anterior pituitary V_1B_ receptor dose‐dependently after oral administration and significantly inhibited the increase in plasma ACTH induced by CRF/desmopressin challenge at doses that showed occupancy of the anterior pituitary V_1B_ receptor of more than 50%. Consistent with these results, THY1773 significantly improved depressive‐like behavior induced by repeated corticosterone injection, a model that induces a depressive state by impairing the HPA axis function (Kamiya et al., 2020), suggesting that THY1773 would be effective for patients with HPA axis hyperactivity, and appropriate for testing its efficacy for MDD.

The accurate prediction of a clinically effective dose is critical for improving efficiency and the success rate in drug development (Zou et al., 2012). The pharmacokinetics/pharmacodynamics (PK/PD) modeling and simulation method is a useful tool for the quantitative decision making on the selection of drug candidates, dosing regimens, and the optimization of study designs, particularly by providing a mechanistic rationale and avoiding inaccuracy caused by interspecies differences. For human PK parameter prediction, many methods have been proposed, and the systemic evaluations of these methods using various data sets of drugs have been reported (Fagerholm, 2007; Lombardo et al., 2013a; Ring et al., 2011). However, there is no perfect method that can predict human PK of all drugs accurately. It is important to select the most appropriate prediction method for each human PK parameter of each drug based on its PK properties. For the efficacy prediction, appropriate translation of preclinical dose‐efficacy findings to human is critical. A target engaging biomarker, such as receptor occupancy (RO), which correlates with efficacy, plays an important role for this translation (Suhara et al., 2017). Establishing an appropriate quantitative model that links PK and target engaging biomarker dynamics is important for the determination of a dose that is predicted to be clinically effective with confidence.

Here, we report our strategy to predict a clinically effective dose of THY1773 for the treatment of MDD to support decision making in the preclinical stage. We applied the methods proposed by Ring et al. (2011) to predict clearance (CL). In addition, as observed human data have become available, the prediction accuracy was retrospectively evaluated, and important insights gained in selecting appropriate methods to successfully predict a clinically effective dose in the preclinical stage are also discussed.

## 2 MATERIALS AND METHODS

### 2.1 Materials

THY1773 and THY1773‐d7, deuterium‐labeled THY1773 used as internal standard (IS), were synthesized at Taisho Pharmaceutical Co., Ltd. Human liver microsomes (HLMs; pooled from 50 donors) were purchased from Sekisui XenoTech, LLC. Recombinant human cytochrome P450 (CYP) enzymes: CYP1A2, CYP2B6, CYP2C8, CYP2C9, CYP2C19, CYP2D6, CYP3A4, and control (Bactosomes, derived from Escherichia coli) were purchased from Cypex Ltd. Pooled plasma of male Crl:CD Sprague Dawley (SD) rat and male beagle dog containing EDTA‐2K as an anticoagulant, were purchased from HAMRI Co., Ltd. Male SD rat and male beagle dog liver microsomes were purchased from Corning Gentest. All other chemicals and solvents were of analytical grade and obtained from commercial sources.

### 2.2 Animals

Male SD rats (7‐week old) were purchased from Charles River Laboratories Japan Inc. Experiments involving male beagle dogs (approximately 3 years; LSG Corporation) were conducted at HAMRI Co., Ltd. The animals were maintained under standard laboratory conditions. All animals were maintained on a standard laboratory animal diet and fasted 16–17 h prior to dosing. Feed was provided 4–5 h postdose and water was allowed ad libitum. All of the animal experimental procedures involving animal handling were approved by the Institutional Animal Care and Use Committee of Taisho Pharmaceutical Co., Ltd (rats and dogs) and HAMRI Co., Ltd (dogs).

### 2.3 In vitro methods

#### 2.3.1 Parallel artificial membrane permeability assay

The membrane permeability of THY1773 was measured using a parallel artificial membrane permeability assay (PAMPA) Evolution instrument (Pion Inc.). A “sandwich” plate consisting of a donor bottom plate with stir bars and an acceptor filter plate was used for the experiment. A donor plate was prepared by the addition of THY1773 to diluted PRISMA HT at pH 6.2 (final concentration of 12.5 μmol/L). An acceptor plate was coated with GIT‐0 lipid solution, followed by the addition of acceptor sink buffer (pH 7.4). After incubation for 4 h at room temperature, the concentrations of THY1773 in the reference, donor, and acceptor solutions were measured with a UV plate reader. The apparent permeability (P_app_) was calculated using the PAMPA Evolution software (version 3.1; Pion Inc.).

#### 2.3.2 Blood‐to‐plasma ratio

The blood‐to‐plasma ratios of THY1773 were analyzed using pooled fresh whole blood (anticoagulant: EDTA‐2K) and control plasma separated from fresh whole blood in parallel from rats, dogs, and humans. Whole blood and control plasma containing 1 μmol/L THY1773 were incubated at 37°C for 30 min in a shaking water bath. The incubated whole blood was centrifuged at 2000 × g for 10 min at 4°C to obtain plasma. An aliquot of each plasma sample (i.e., isolated plasma from incubated whole blood and control plasma) was mixed with 8 volumes of acetonitrile/methanol (9:1, vol/vol) containing the IS. After centrifugation at 3974 × g for 10 min at 4°C, the supernatant was subjected to liquid chromatography/tandem mass spectrometry (LC‐MS/MS) analysis. The blood‐to‐plasma ratio was calculated by dividing the THY1773 concentration in control plasma, which is the same as the concentration in whole blood, by that in the separated plasma from incubated whole blood.

#### 2.3.3 Plasma protein binding

The plasma protein binding of THY1773 in rats, dogs, and humans was evaluated by an equilibrium dialysis method using a 96‐well equilibrium dialysis plate (HTDialysis) with a 12–14 kDa cut‐off dialysis membrane. THY1773 was dissolved in dimethyl sulfoxide, and spiked with rat, dog, or human plasma at a final concentration of 300 ng/ml. An aliquot of plasma containing THY1773 and sodium phosphate buffer (pH 7.4) was added into the donor side and the receiver side of each designated well, respectively. After incubation for 6 h at a rate of 50 oscillation/min at 37°C in an air incubator, an aliquot of each side of each well was collected and mixed with acetonitrile/methanol (9:1, vol/vol) containing the IS. Each sample was centrifuged at 3974 × g for 10 min at 4°C to remove the precipitated proteins, and the resultant supernatants were subjected to LC‐MS/MS analysis. The protein binding (%) was calculated using the Boudinot formula (Boudinot & Jusko, 1984).

#### 2.3.4 Metabolic stability in animal liver microsomes

The oxidative metabolism of THY1773 was evaluated in rat and dog liver microsomes. THY1773 (1 μmol/L) was incubated with pooled liver microsomes (0.25 mg protein/ml) in sodium–potassium phosphate buffer (pH 7.4) consisting of 100 mmol/L sodium phosphate, 100 mmol/L potassium chloride, 2.5 mmol/L magnesium chloride (MgCl_2_), 1.5 mmol/L glucose‐6‐phosphate (G‐6‐P), and 0.18 units/ml G‐6‐P dehydrogenase (G‐6‐PDH). The reactions were initiated by the addition of *β*‐nicotinamide–adenine dinucleotide phosphate, oxidized form (NADP^+^, 0.16 mmol/L), and incubated for 10, 20, 30, 40, 50, and 60 min. The reactions were terminated by the addition of an equal volume of acetonitrile/methanol (9:1, vol/vol) containing the IS, followed by centrifugation at 3974 × g for 10 min at 4°C. An aliquot of the supernatant was subjected to LC‐MS/MS analysis. The intrinsic clearance (CL_int_) was calculated by the substrate depletion method described by Naritomi et al. (2001).

#### 2.3.5 Reaction phenotyping

Reaction phenotyping using cDNA expressed recombinant human CYP isoforms, CYP1A2, CYP2B6, CYP2C8, CYP2C9, CYP2C19, CYP2D6, and CYP3A4, was conducted. Each recombinant enzyme (100 pmol/ml) in potassium phosphate buffer (pH 7.4; 100 mmol/L for CYP3A4, 50 mmol/L for the others) was preincubated with [^3^H]THY1773 (10 μmol/L) at 37°C for 5 min. The reactions were initiated by the addition of NADPH‐generating system (1 mmol/L NADP^+^, 5 mmol/L G‐6‐P, 1 unit/ml G‐6‐PDH, and 5 mmol/L MgCl_2_) and incubated for 0 or 60 min. The reactions were terminated by the addition of an equal volume of acetonitrile/methanol (9:1, vol/vol), followed by centrifugation at 1650 × g for 10 min at 4°C. An aliquot of the supernatant was subjected to an Agilent 1200 HPLC system (Agilent Technologies, Inc.) equipped with a radiochemical flow detector (Radiomatic 625TR; Perkin Elmer, Inc.), and formation of metabolites was evaluated. Reaction phenotyping study was also conducted using HLMs and selective chemical inhibitors for CYP2D6 and CYP3A, which were assumed to have significant roles in THY1773 metabolism from recombinant metabolic stability. Quinidine (1 μmol/L) for CYP2D6 and ketoconazole (1 μmol/L) for CYP3A were used as selective inhibitors. THY1773 (500 nmol/L) was incubated with pooled HLMs (0.5 mg protein/ml) in potassium phosphate buffer (100 mmol/L, pH 7.4) in the absence or presence of the selective inhibitors. The reactions were initiated by the addition of NADPH generating system (1.3 mmol/L *β*‐NADP^+^, 3.3 mmol/L G‐6‐P, 3.3 mmol/L MgCl_2_, and 0.4 units/ml G‐6‐PDH), and incubated at 37°C for 30 min. The reactions were terminated by the addition of two volumes of acetonitrile/methanol (9:1, vol/vol) containing the IS. After centrifugation at 3974 × g for 10 min at 4°C, an aliquot of the supernatant was subjected to LC‐MS/MS analysis. The CL_int_ was calculated by the method as described previously under metabolic stability in animal liver microsomes, and the fraction metabolized (fm) was calculated by Equation (1):

(1) $$\text{fm}=1-\left({\text{CL}}_{\text{int, with inh}}{\text{/CL}}_{\text{int}}\right)$$

where CL_int_ is intrinsic clearance without specific inhibitor and CL_int,with inh_ is intrinsic clearance in the presence of specific inhibitor.

### 2.4 PK in rats and dogs

The PK and urinary excretion of THY1773 in rats (1 mg/kg) and dogs (0.5 mg/kg) under fasted conditions were evaluated following intravenous or oral administration of THY1773. THY1773 was dissolved in 10% (vol/vol) hydroxypropyl‐*β*‐cyclodextrin at pH 4 for intravenous administration, or suspended in 0.5% (wt/vol) methyl cellulose 400 for oral administration. After a single intravenous or oral administration of THY1773, blood samples were collected into tubes containing EDTA‐2K as an anticoagulant. The scheduled time points were predose (dog only), 5 (intravenous only), 15, 30 min, 1, 2, 4, 8, 12, and 24 h postdose. Plasma was separated by centrifugation. Urine samples were collected into bottles set on ice for 24 h after intravenous administration of THY1773. Plasma and urine samples were treated with 4 volumes of acetonitrile/methanol (9:1, vol/vol) containing the IS. After centrifugation (3639 × g, 4°C, 10 min) to remove precipitated proteins, the supernatant was subjected to LC‐MS/MS analysis. The distributions of THY1773 into brain and pituitary gland in rats were investigated after a single oral administration of THY1773 (1 mg/kg). Blood was collected from the tail vein into tubes containing EDTA‐2K at 1 h after a single oral administration of THY1773, and the plasma was separated by centrifugation (12000 × g, 4°C, 3 min). After blood collection, the brain and pituitary gland were excised followed by homogenization with approximately 4 volumes of distilled water. Plasma, brain, and pituitary samples were treated with 4 volumes of acetonitrile/methanol (9:1, vol/vol) containing the IS and centrifuged (3639 × g, 4°C, 10 min). Aliquots of the supernatant were subjected to LC‐MS/MS analysis.

### 2.5 PK analysis

The plasma concentration–time profiles of THY1773 were analyzed by a non‐compartmental analysis with PK analysis software Phoenix WinNonlin® (version 6.1; Certara), and the PK parameters were calculated. The fraction excreted in urine was calculated by dividing the amount of THY1773 excreted in urine by the administered dose.

### 2.6 Prediction of human PK parameters

#### 2.6.1 CL

Of the many reported methods for the prediction of human CL, the top two methods reported by Ring et al. (2011) were selected based on prediction accuracy; that is, the unbound fraction corrected intercept method (FCIM) and two‐species‐based allometry (rat and dog) described by Equations (2) and (3), respectively (Tang, Hussain, Leal, Mayersohn, & Fluhler, 2007; Tang & Mayersohn, 2005):

(2) $${\text{CL}}_{\text{human}}\left(\text{mL/min/kg}\right)=33.35\times {\left(a/Rf_{u}\right)}^{0.77}/\text{Human}\;\text{body}\;\text{weight}\;\left(\text{kg}\right)$$

(3) $$\text{CL}=a\times {\left(\text{Human}\;\text{body}\;\text{weight}\right)}^{0.628}$$

where the allometric coefficient (a) is obtained from the intercept of the simple allometry log–log plot between rats and dogs, and Rf_u_ is the ratio of unbound fraction in plasma between rats and humans. Human body weight was assumed to be 70 kg. The hepatic CL (CL_liver_) was also predicted by physiologically based in vitro–in vivo extrapolation (IVIVE) method using physiological parameters listed in Table 1 based on the well stirred model (Yang, Jamei, Yeo, Rostami‐Hodjegan, & Tucker, 2007).

**TABLE 1:** Physiological parameters used for in vitro–in vivo extrapolation

Species	Liver weight per body weight^a^ (g/kg)	Microsomal protein per liver weight (mg/g)	Hepatic blood flow (ml/h/kg)	Glomerular filtration rate^b^ (ml/h/kg)
Rat	40	45^a^	4200^a^	312
Dog	32	45^a^	2400^a^	192
Human	21	40^c^	1242^c^	108

^a^ Hosea et al. (2009).

^b^ Paine et al. (2011).

^c^ Brown et al. (2007).

Since the CL_liver_ was underestimated in rats and dogs from CL_int_ in liver microsomes, the ratio of observed in vivo CL_int_ and predicted CL_int_ was calculated as the empirical scaling factor (ESF) for the extrapolation of CL_liver_ from in vitro data. Human CL_liver_ was predicted from CL_int_ in HLMs with and without applying the average of ESF derived from rats and dogs. The total plasma clearance (CL_total_) was calculated by Equation (4):

(4) $${\text{CL}}_{\text{total}}=\;\frac{Q_{H}\times f_{u}\times \left({\text{CL}}_{\text{int}}/f_{\text{u,mic}}\right)}{Q_{H}+\left(f_{u}/R_{b}\right)\times \left({\text{CL}}_{\text{int}}/f_{\text{u,mic}}\right)}+{\text{CL}}_{\text{renal}}$$

where Q_H_ is hepatic blood flow, f_u_ is the free fraction in plasma, f_u,mic_ is the fraction unbound in HLMs, R_b_ is blood‐to‐plasma ratio, and CL_renal_ is renal clearance. The f_u,mic_ value was calculated using the Simcyp population based simulator (Version 16 R1; Simcyp Ltd.). The CL_renal_ was estimated by multiplying f_u_ by glomerular filtration rate (GFR), assuming glomerular filtration only.

#### 2.6.2 Volume of distribution

Human volume of distribution at steady state (Vd_ss_) was predicted by single species scaling from rat assuming that the unbound Vd_ss_ is comparable across species, as described in Equation (5):

(5) $${\text{Vd}}_{\text{ss,human}}=\left(\frac{{\text{Vd}}_{\text{ss,rat}}}{f_{\text{u,rat}}}\right)\times f_{\text{u,human}}$$

where f_u_ is the unbound fraction in plasma. Human Vd_ss_ was also predicted by the two methods reported to be the best methods based on the prediction accuracy (Jones et al., 2011), which were Øie‐Tozer method (Obach et al., 1997) and rat–dog–human proportionality equation described (Wajima, Fukumura, Yano, & Oguma, 2003) by Equations (6) and (7), respectively:

(6) $${\text{Vd}}_{\text{ss}}\left[\text{L/kg}\right]=V_{p}\times \left(1+R_{\text{E/I}}\right)+V_{p}\times f_{u}\times \left(V_{e}/V_{p}-R_{\text{E/I}}\right)+V_{r}\times f_{u}/f_{\text{u,t}}$$

(7) $$\log\left({\text{Vd}}_{\text{ss,human}}\right)=0.07714\times \log\left({\text{Vd}}_{\text{ss,rat}}\right)\times \log\left({\text{Vd}}_{\text{ss,dog}}\right)+0.5147\times \log\left({\text{Vd}}_{\text{ss,dog}}\right)+0.586$$

where f_u,t_ is the fraction unbound in tissues, R_E/I_ is the ratio of binding protein in the extracellular fluid to that in plasma, V_e_, V_p_, and V_r_ is the extracellular, plasma, and remaining fluid volume, respectively. Volumes and plasma protein levels were used as described by Obach et al. (1997).

### 2.7 Physiologically based pharmacokinetics/RO modeling and simulation

#### 2.7.1 PBPK/RO model development

The human PBPK/RO models were developed using Simcyp simulator following the previously reported strategy (Jones, Parrott, Jorga, & Lavé, 2006). Since Vd_ss_ in rat was underpredicted using tissue‐to‐plasma partition method as proposed by Rodgers and Rowland (2007), the tissue‐to‐plasma partition coefficient (Kp) scalar of 0.422 was applied to all human tissue compartments to match the predicted human Vd_ss_ by the Øie‐Tozer method. The CL_int_ in HLMs were inputted into Simcyp as CYP2D6 and CYP3A4 dependent CL_int_ based on the estimated fraction metabolized in HLMs, with (Model 1) or without (Model 2) applying ESF. The predicted CL_renal_ assuming glomerular filtration only was also inputted. As the relationship between drug concentration and RO typically follow the E_max_ model (Kim et al., 2012), the E_max_ model described by Equation (8) was used as a PD model for THY1773 V_1B_ RO. As the pituitary gland is outside of the blood–brain barrier, the free concentration of THY1773 around the V_1B_ receptor in the pituitary gland was assumed to be equal to the free concentration in plasma:

(8) $$\text{RO}\;\left(\%\right)=\left(E_{\text{max}}\times C_{\text{p,u}}\right)/\left({\text{EC}}_{50}+C_{\text{p,u}}\right)$$

where C_p,u_ is free concentration in plasma, E_max_ is the maximum V_1B_ RO, and EC_50_ is plasma free concentration required to produce 50% of E_max_. E_max_ was assumed to be 100%, and EC_50_ was assumed to be comparable to the inhibition constant (K_i_) calculated from the in vitro binding assay, which evaluated the binding of [^3^H]AVP to the membranes of 1321‐N1 cells expressing the human V_1B_ receptor with or without 0.001–1000 nmol/L of THY1773 by a previously reported method (Iijima et al., 2014). The input parameters used for human PBPK/RO model development in Simcyp are listed in Table 2.

**TABLE 2:** Human PBPK/RO model input parameters for THY1773

Parameter	Value	Reference/comment	
Physicochemical properties			
Molecular weight	483.99		
Log P	3.17	Experimental data	
Compound type	Monoprotic base		
pKa	6.85	Experimental data	
Fraction unbound in plasma	0.017	Experimental data	
Blood‐to‐plasma ratio	0.55	Theoretical limit (experimental data: 0.54)	
Absorption			
Absorption model	Advanced dissolution, absorption, and metabolism model		
Fraction absorbed	0.998	Simcyp predicted	
Absorption rate constant (1/h)	3.10	Simcyp predicted	
PAMPA permeability (10^−6^ cm/s)	128.9	Experimental data	
Fraction unbound in gut	1	Assumption
Distribution		
Distribution model	Full PBPK	
Vd_ss_ (L/kg)	0.405	Adjusted to predicted value by Øie‐Tozer method with tissue to plasma partition coefficient scalar (0.422)
Elimination		
In vitro metabolic system	HLMs enzyme kinetics	
CL_int_ (μl/min/mg protein)	11.2 (Model 1)	Experimental data
	56.4 (Model 2)	Experimental data with empirical scaling factor
f_m,CYP2D6_/f_m,CYP3A4_	0.089/0.911	Experimental data
Fraction unbound in microsomes	0.714	Simcyp predicted
Renal clearance (L/h)	0.133	Estimated by multiplying f_u_ and mean body weight of “Sim‐Healthy Volunteers” population (72.4 kg) by GFR
Pharmacodynamic (receptor occupancy) model		
Model	Simple E_max_ model	
E_max_ (%)	100	Assumption
EC_50_ (nmol/L)	3.51	Assumption (K_i_ value for human V_1B_ receptor binding inhibition)

GFR, glomerular filtration rate; PAMPA, parallel artificial membrane permeability assay; PBPK, physiologically based pharmacokinetics; RO, receptor occupancy.

#### 2.7.2 Prediction of a clinically effective dose

The plasma concentration– and RO–time profiles of THY1773 after oral administration of various doses of THY1773 were simulated by the previously described PBPK/RO model in 10 virtual trials of 10 healthy volunteers (ages 20–50 years, male/female ratio of 50/50) assuming linear PK. The built‐in “Sim‐Healthy Volunteers” population was used. According to the results of a CRF/desmopressin challenge study and a forced swim test in rats, significant effects for both studies were shown at doses which showed more than 50% occupancy of the anterior pituitary V_1B_ receptor (Kamiya et al., 2020). Therefore, the clinically effective dose was assumed to be the dose that maintains RO at more than 50% for the dosing interval (24 h after administration).

### 2.8 Phase 1 clinical study

A first‐in‐human, randomized, double‐blind, placebo‐controlled, adaptive, single‐ascending dose study was conducted to evaluate the safety, tolerability, and PK of single oral doses of TS‐121 capsules in the United States. This study was conducted in accordance with the ethical principles of the Declaration of Helsinki, International Council for Harmonization Good Clinical Practice Guidelines, and the United States Food and Drug Administration Code of Federal Regulations. The study was approved by an institutional ethical review board. All subjects provided written informed consent before participating in the study. Sixty‐eight healthy male or female volunteers were enrolled and received a single oral dose of either TS‐121 capsules (0.5, 2.5, 4, 8, 15, 30, or 50 mg as THY1773/day) or placebo in a 3:1 ratio in a fasted state.

Blood samples were collected at predose and 0.25, 0.5, 0.75, 1, 1.5, 2, 3, 4, 6, 8, 10, 12, 24, 36, and 48 h postdose. Urine samples were collected before dosing and at the following time intervals after dosing: 0–6, 6–12, 12–24, 24–36, and 36–48 h. THY1773 plasma and urine concentrations were determined by validated LC‐MS/MS methods. PK parameters were evaluated following the method as described in Section 2.5 PK analysis.

### 2.9 Evaluation of prediction accuracy

The fold error of the difference between observed and predicted human PK parameters was calculated by dividing the predicted value by the observed value. The bioavailability of THY1773 was assumed to be 100%, and the volume of distribution at steady state was assumed to be comparable to that during the terminal phase.

### 2.10 LC‐MS/MS conditions

The LC‐MS/MS system consisted of a Shimadzu LC‐10ADvp, LC‐20AD, LC‐30AD, or Nexera (Shimadzu) and an API 3000, API 4000, or Triple Quad 5500 mass spectrometer (AB Sciex). The data were collected and processed using Analyst software (version 1.4 or later; AB Sciex). THY1773 was analyzed using a Shim‐pack XR‐ODS column (3.0 mm I.D. × 30 mm, 2.2 μm particle size; Shimadzu) or a ZORBAX Eclipse Plus C18 column (4.6 mm I.D. × 50 mm, 3.5 μm particle size; Agilent Technologies) with water/formic acid (1000:1, vol/vol) and acetonitrile or water/formic acid (95:5, vol/vol) and acetonitrile/methanol (95:5, vol/vol) as the mobile phase under a gradient condition. The selected reaction monitoring transitions were as follows: THY1773, m/z 484 → m/z 397; and THY1773‐d7 (IS), m/z 491 → m/z 404.

## 3 RESULTS

### 3.1 Permeability, plasma protein binding, and blood‐to‐plasma ratio

The passive membrane permeability of THY1773 was measured using PAMPA. The P_app_ of THY1773 in PAMPA at pH 6.2 was 128.9 (10^−6^ cm/s), suggesting high permeability in the gut. Plasma protein binding was determined by equilibrium dialysis at a THY1773 concentration of 300 ng/ml, which was roughly estimated as an average concentration in human plasma at an effective dose. The mean plasma protein binding of THY1773 at 300 ng/ml was 89.2% in rats, 89.6% in dogs, and 98.3% in humans. The blood‐to‐plasma ratio of THY1773 was evaluated in rat, dog, and human pooled whole blood at 1 μmol/L (484 ng/ml). The mean blood‐to‐plasma ratio was 0.822 in rats, 0.846 in dogs, and 0.540 in humans. THY1773 showed higher plasma protein binding and lower blood‐to‐plasma ratio in humans than in animals.

### 3.2 Metabolic stability in animal liver microsomes

The oxidative metabolism of THY1773 in rat and dog liver microsomes was evaluated at 1 μmol/L, at which the reaction was assumed to be linear. The CL_int_ (in units of μL/min/mg protein) in liver microsomes was 22.0 for rats and 32.2 for dogs.

### 3.3 Reaction phenotyping

Reaction phenotyping studies were conducted using cDNA expressed recombinant human CYP isoforms and HLMs. In the reaction phenotyping study using recombinant human CYP isoforms, [^3^H]THY1773 metabolite formation was evaluated at 10 μmol/L. [^3^H]THY1773 was metabolized by CYP2D6 and CYP3A4, and to a minor extent by CYP2C8. In the reaction phenotyping study using HLMs and selective chemical inhibitors, the disappearance of THY1773 at 500 nmol/L (242 ng/ml) was evaluated. The CL_int_ of THY1773 was significantly reduced by ketoconazole, a CYP3A inhibitor, whereas it was partially reduced by quinidine, a CYP2D6 inhibitor. The fractions metabolized were estimated to be 0.914 by CYP3A and 0.0893 by CYP2D6 (Table 3).

**TABLE 3:** Effects of specific inhibitor of CYP3A and CYP2D6 on CL_int_ of THY1773 in human liver microsomes and estimated fraction metabolized

CYP isoforms	Selective inhibitor (concentration)	CL_int_ (μl/min/mg)	f_m_
–	None	11.2 ± 0.898	N.A.
CYP3A	Ketoconazole (1 μmol/L)	0.961^a^	0.914
CYP2D6	Quinidine (1 μmol/L)	10.2 ± 0.509	0.0893

Values are presented as the mean ± SD of triplicate determinations.

N.A., not applicable.

^a^ Value is presented as the mean of duplicate determinations.

### 3.4 PK in rats and dogs

**FIGURE 1:**
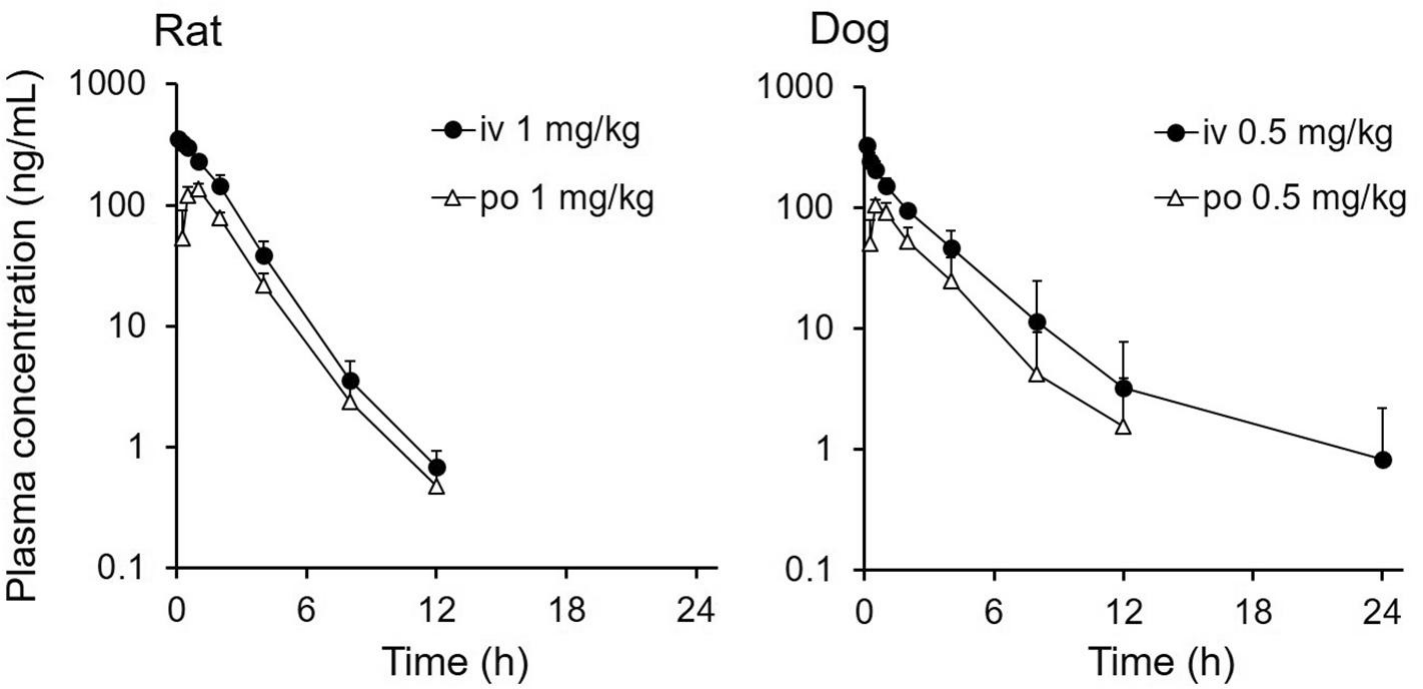
THY1773 plasma concentration–time profiles (mean ± SD, n = 3) in male rats (left) and dogs (right) after a single intravenous (iv) or oral (po) administration. The plasma concentrations declined below the lower limit of quantification (<0.1 ng/ml) at 24 h except for after intravenous dose to dogs.

The concentration–time profiles in rats and dogs after a single intravenous or oral administration of THY1773 under fasted conditions are shown in Figure 1, and the PK parameters are summarized in Table 4. In rats and dogs, the CL_total_ was moderate (approximately 30% of hepatic blood flow) after single intravenous administration. The urinary excretion of unchanged THY1773 within 24 h postintravenous dosing was 4.6% in rats and 2.0% in dogs. In both species, the CL_renal_ of THY1773 was comparable to f_u_ × GFR (within twofold), which suggested that active transport was not likely to play an important role in the CL_renal_. Following oral administration, THY1773 was absorbed rapidly, and oral bioavailability reached 46.4% in rats and 45.0% in dogs. The concentration ratio of brain and pituitary gland to plasma in rats at 1 h after oral administration was 0.2 and 4.7, respectively.

**TABLE 4:** Pharmacokinetic parameters of THY1773 after a single intravenous or oral administration to male rats (1 mg/kg) and dogs (0.5 mg/kg)

Species	Route	CL_total_ (ml/h/kg)	Vd_ss_ (ml/kg)	t_1/2_ (h)	C_max_ (ng/ml)	t_max_ (h)	AUC_0‐∞_ (h·ng/ml)	F (%)
Rat	iv	1330 (13.7)	2220 (5.7)	1.38 (2.3)	N.C.	N.C.	762 (14.9)	
	po	N.C.	N.C.	1.45 (5.5)	136 (11.8)	0.833 (34.6)	355 (13.5)	46.6
Dog	iv	785 (26.4)	2020 (37.6)	3.07 (59.3)	N.C.	N.C.	664 (23.7)	
	po	N.C.	N.C.	2.40 (81.2)	105 (12.3)	0.500 (0.0)	307 (37.7)	45.0 (16.6)

Values are presented as the mean (percent coefficient of variation) of three animals.

N.C., not calculated.

### 3.5 Prediction of human PK parameters

Of many reported methods for the prediction of human PK parameters, the methods reported to be relatively accurate among various methods using data sets containing lipophilic and highly plasma protein‐bound drugs (Jones et al., 2011; Ring et al., 2011) were selected, as THY1773 was also lipophilic and highly plasma protein‐bound. The human CL_total_ was predicted by FCIM, two‐species‐based allometry, or IVIVE method (Table 5). Before conducting IVIVE, the discrepancy between in vitro and in vivo CL_int_ was evaluated in rats and dogs. The in vivo CL_int_ was higher than in vitro CL_int_ in both species, and the ESF for IVIVE was calculated to be 6.5 in rats and 3.6 in dogs. The predicted human CL_total_ values varied greatly depending on the prediction methods, and the difference was over 14‐fold. The human Vd_ss_ was predicted by rat single species scaling with Equation (5), Øie‐Tozer method, or rat–dog–human proportionality equation (Table 6). The predicted Vd_ss_ values varied depending on the prediction methods, and the difference was over threefold.

**TABLE 5:** Predicted and observed human CL_total_ of THY1773 and fold error

Prediction method	Predicted human CL_total_ (ml/h/kg)	Observed human CL_total_/F (ml/h/kg)	Fold error^a^
FCIM	64.1	17.2	3.7
Two species allometry	224		13
IVIVE method without ESF	15.0		0.87
IVIVE method with ESF	63.4		3.7

ESF, empirical scaling factor; FCIM, fraction corrected intercept method; IVIVE, in vitro–in vivo extrapolation.

^a^ Fold error was calculated by dividing predicted CL_total_ by observed oral CL_total_ (CL_total_/F) assuming bioavailability (F) was 100%.

**TABLE 6:** Predicted and observed human Vd_ss_ of THY1773 and fold error

Prediction method	Predicted human Vd_ss_ (ml/kg)	Observed human Vd_z_/F (ml/kg)	Fold error^a^
Single species scaling (rat)	349	528	0.66
Rat–dog–human proportionality equation	1380		2.6
Øie‐Tozer method	404		0.77

^a^ Fold error was calculated by dividing predicted Vd_ss_ by observed oral apparent volume of distribution during terminal phase (Vd_z_/F) assuming bioavailability (F) was 100% and the volume of distribution at steady state was comparable to that during terminal phase.

### 3.6 Prediction of a clinically effective dose

**FIGURE 2:**
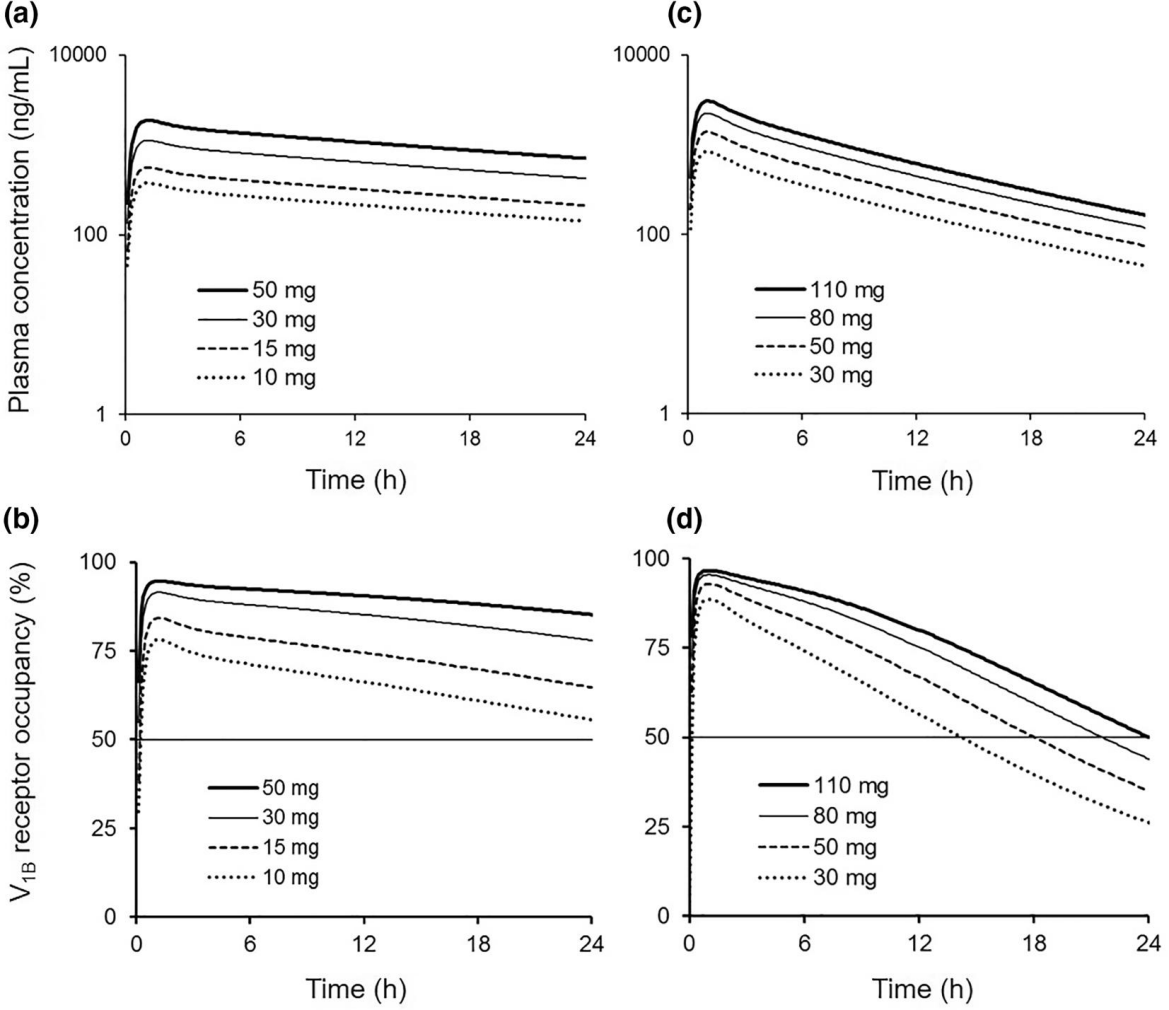
The simulated plasma THY1773 concentration–time (a and c) and V_1B_ RO–time (b and d) profiles in humans after a single oral administration of THY1773 at various doses (10, 15, 30, and 50 mg for Model 1; 30, 50, 80, and 110 mg for Model 2) using the human PBPK/RO model. The PBPK/RO model was established using CL_int_ in HLMs without ESF (Model 1; a and b) or with ESF (Model 2; c and d). The solid horizontal lines represent 50% RO. ESF, empirical scaling factors; HLM, human liver microsome; PBPK, physiologically based pharmacokinetics; RO, receptor occupancy

The plasma concentration– and RO–time profiles in humans were simulated by two PBPK/RO models considering best and worst case scenarios for human liver CL_int_. The simulated plasma concentration– and RO–time profiles in humans at various doses of THY1773 using PBPK/RO model (Model 1 as best and Model 2 as worst case scenario) are shown in Figure 2. As described in Section 2.7.2, preclinical studies indicated that THY1773 exhibits antidepressant‐like effects in several animal models at doses that occupied more than 50% of the pituitary V_1B_ receptor; consequently, we assumed that significant efficacy would be shown at doses that maintain more than 50% occupancy of the V_1B_ receptor for the dosing interval (24 h after administration). Based on our assumption, the clinically effective dose was predicted to be 10 mg by Model 1 and 110 mg by Model 2.

### 3.7 Phase 1 clinical study

**FIGURE 3:**
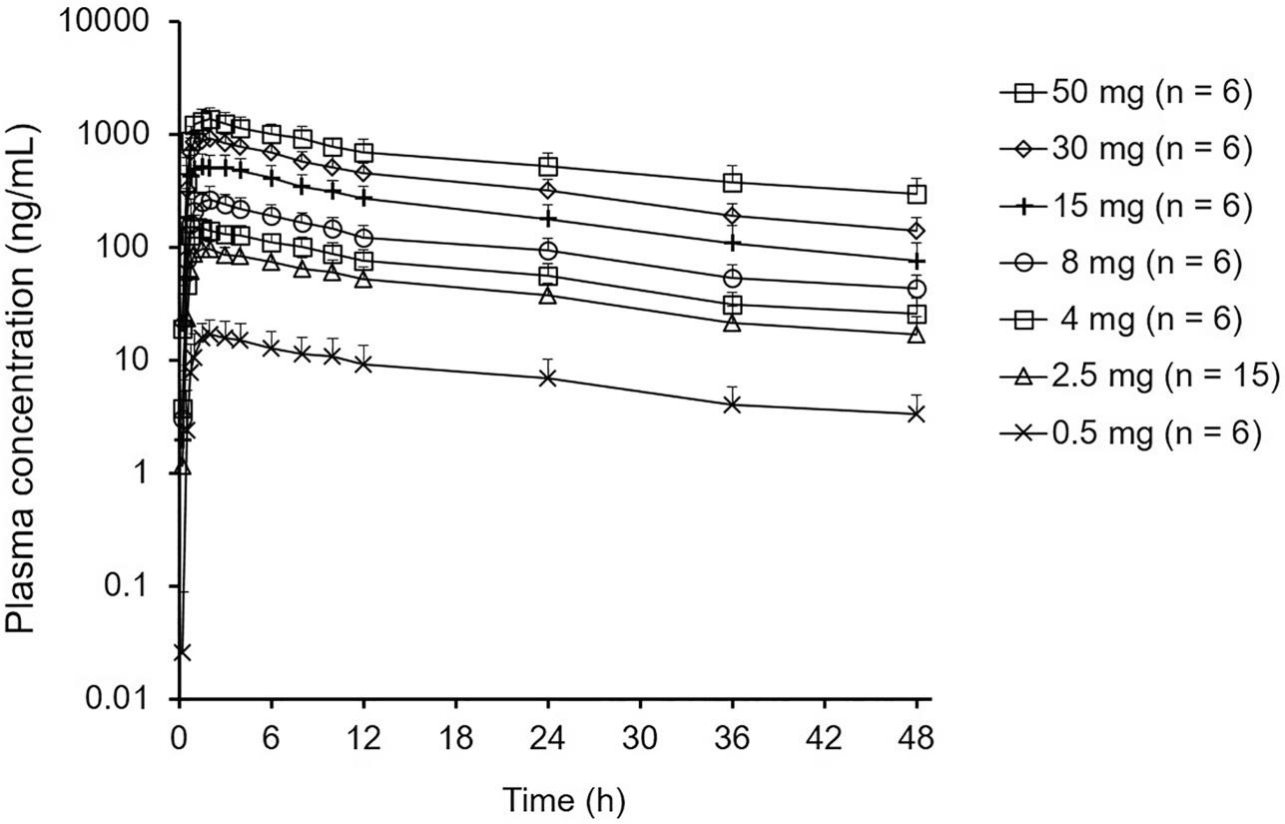
The plasma concentration–time profiles (mean ± SD) of THY1773 after oral administration in the phase 1 single ascending dose study. Healthy male or female volunteers received a single oral dose of either TS‐121 capsules or placebo in a fasted state.

Following a single oral administration of TS‐121 capsules in healthy male or female volunteers, THY1773 was absorbed rapidly, reached maximum plasma concentration (C_max_) at 1.3 h, and eliminated with a half‐life of 21 h at 30 mg/day as THY1773 (Figure 3 and Table 7). THY1773 exposures (C_max_ and area under the concentration–time curve) increased nearly dose‐proportionally (Figure 3). Oral CL_total_ and apparent volume of distribution during the terminal phase were stable over the dose range of 0.5 to 50 mg, and averaged 1.32 L/h and 40.6 L, respectively. The urinary excretion of unchanged THY1773 within 48 h postoral dosing was also stable over the dose range and averaged 4.2%.

**TABLE 7:** Observed human PK parameters of THY1773 after a single oral administration (30 mg)

Parameter	AUC_0‐∞_ (h·ng/ml)	C_max_ (ng/ml)	t_max_ ^a^ (h)	t_1/2_ (h)	CL_po_ (L/h)	Vd_z_/F (L)
Mean	21340	949	1.27	20.5	1.52	43.6
(% CV)	(24.2)	(22.4)	(1.00, 2.10)	(14.4)	(37)	(27.3)

^a^ Median (minimum, maximum) values are presented.

### 3.8 Evaluation of prediction accuracy

**FIGURE 4:**
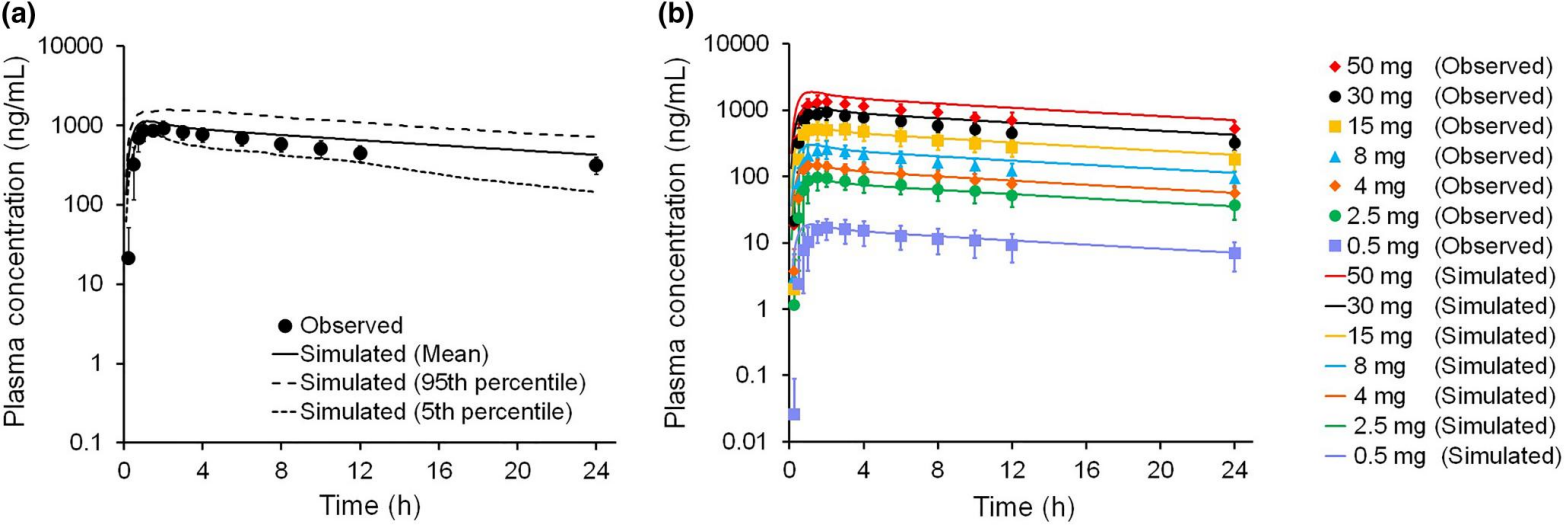
The simulated versus observed plasma concentration–time profiles of THY1773 in humans after a single oral administration of THY1773 at a dose of 30 mg (a) and at doses of 0.5–50 mg (b) using the PBPK model. The PBPK model was established using CL_int_ in HLMs without ESF (Model 1). Points, observed values (mean ± SD) in clinical study; solid line, simulated mean; dotted lines, 5th and 95th percentiles. ESF, empirical scaling factors; HLM, human liver microsome; PBPK, physiologically based pharmacokinetics

The observed oral blood CL was only 2.6% of the hepatic blood flow, which indicated that hepatic metabolism of THY1773 was not extensive in humans. As THY1773 was mainly metabolized by CYP3A in HLMs, we assumed that gut metabolism, which was known to be mainly mediated by CYP3A, was not extensive in humans either. Then we assumed that the human bioavailability of THY1773 was 100% to compare the predicted human PK parameters with the observed values obtained after oral administration. The fold errors of the difference between observed and predicted human PK parameters are shown in Table 5 (CL_total_) and Table 6 (Vd_ss_). The simulated concentration–time profiles of THY1773 using PBPK model (Model 1) overlaid with observed plasma concentrations after a single oral administration of THY1773 at a dose of 30 mg and at doses of 0.5–50 mg are shown in Figure 4. Overall, concentrations of THY1773 were accurately predicted by Model 1, whereas they were underestimated by Model 2.

## 4 DISCUSSION AND CONCLUSION

The development of drugs for central nervous system (CNS) diseases is challenging relative to other therapeutic areas (Harrison, 2016; Suhara et al., 2017). Modeling and simulation technology has been used widely as an initiative to overcome the difficulty and to improve success rates of drug discovery and development (Visser, De Alwis, Kerbusch, Stone, & Allerheiligen, 2014). We developed THY1773, a novel selective V_1B_ receptor antagonist that exhibited antidepressant‐like effects in animal models (Kamiya et al., 2020). In the present studies, we established PBPK/RO models of THY1773 fully based on the preclinical data and predicted a clinically effective dose for MDD to support decision making in the preclinical stage of drug development.

In rats and dogs, THY1773 was rapidly and well absorbed with moderate bioavailability, and exhibited moderate CL_total_ and Vd_ss_. The renal excretion ratio was less than 5%. As THY1773 had high permeability in PAMPA (a class 2 compound in the extended CL classification system; Varma, Steyn, Allerton, & El‐Kattan, 2015), THY1773 was assumed to be primarily eliminated by hepatic metabolism. THY1773 showed species differences in plasma protein binding and blood‐to‐plasma ratio. THY1773 is a CYP3A4 substrate, and mainly metabolized by CYP3A in HLMs. The CL_int_ in HLMs was lower than that in rats and dogs. From these preclinical PK properties, THY1773 was expected to be well absorbed with good bioavailability and eliminated with moderate CL mainly mediated by CYP3A4 metabolism in humans. Based on these preclinical data, a PBPK/RO model of THY1773 in humans was established for the prediction of a clinically effective dose for MDD.

First, human CL and Vd_ss_ were predicted by several methods. Human CL was predicted by two allometric and two IVIVE methods, and we found that widely different values were obtained depending on the methods. Of these methods, we selected IVIVE methods for the following reasons: (1) the plasma protein binding of THY1773 was higher in humans than in animals, and it has been reported that allometric methods that did not account for protein binding tended to overpredict human CL (Ring et al., 2011); and (2) as THY1773 is a CYP3A4 substrate, the CL_liver_, or intestinal first pass, was expected to be relatively accurately predicted using CL_int_ in HLMs (Bowman & Benet, 2019b; Gertz, Harrison, Houston, & Galetin, 2010). Since IVIVE methods using HLMs reportedly have a tendency to underpredict human CL_liver_ (Wood, Houston, & Hallifax, 2017), the IVIVE method, with or without ESF obtained from rats and dogs, was used for THY1773 CL_liver_ prediction as the worst or best case scenario, respectively. To predict CL_total_, CL_renal_ was predicted assuming glomerular filtration only as in animals, and added to the predicted CL_liver_. The human Vd_ss_ was predicted by two empirical and one semi‐mechanistic method. The predicted values were different depending on whether or not the plasma protein binding was considered. The unbound Vd_ss_ was comparable between rats and dogs, suggesting that the assumption in rat single species scaling with Equation (5), (i.e., the unbound Vd_ss_ is comparable across species), was valid, and the predicted human Vd_ss_ would therefore be confident. We ultimately selected the semi‐mechanistic Øie‐Tozer method as it was reported to be most accurate for many compounds (Jones et al., 2011), and the predicted Vd_ss_ was comparable to that predicted by rat single species scaling with Equation (5).

Then, the PBPK model was established using preclinical data, adjusting the Vd_ss_ to fit the predicted value by the Øie‐Tozer method, and linked to the RO. The following two assumptions were made: (1) the pituitary V_1B_ RO of THY1773 was correlated to the plasma free concentration, and the relationship follows E_max_ model, and (2) the in vivo EC_50_ value for pituitary V_1B_ RO was comparable to the in vitro K_i_ value for human V_1B_ receptor binding inhibition.

Finally, a clinically effective dose was predicted by the PBPK/RO model assuming that significant efficacy would be shown at a dose that shows more than 50% occupancy of the V_1B_ receptor for the dosing interval. As a result, the predicted effective dose of THY1773 was relatively low (10 mg/day, or at maximum, 110 mg/day), and was thought to be optimal for further clinical development. These results supported our decision to enter phase 1 clinical trials.

In the phase 1 single ascending dose study, THY1773 was rapidly absorbed, and eliminated with a terminal half‐life of approximately 21 h, which was long enough to support once daily dosing. THY1773 demonstrated linear PK over the dose range of 0.5–50 mg. Together with the additional information that THY1773 did not have any apparent in vitro inhibition or induction potential on major CYP isoforms (data not shown), THY1773 demonstrated ideal PK features as a CNS drug. Plasma concentration–time profiles of THY1773 after a single oral administration were consistent with the simulated profiles by PBPK model without applying ESF. Based on the phase 1 RO study using PET (ClinicalTrials.gov Identifier: NCT02448212), V_1B_ RO at steady state was estimated to be 55.3% at 10 mg and 75.1% at 50 mg (unpublished data), which were comparable to our prediction (68.2% at 10 mg and 90.3% at 50 mg) and expected to be enough to show efficacy. In our phase 2 study conducted in patients with MDD (ClinicalTrials.gov Identifier: NCT03093025), statistically nonsignificant greater improvements in depressive symptoms were observed with TS‐121 in MDD patients at doses of 10 and 50 mg, which would achieve more than 50% RO at steady state (Kamiya et al., 2020). Although further investigation of the dose‐efficacy relationship is required, these findings support our assumption that a clinically effective dose that maintains RO at more than 50% for the dosing interval is achievable.

A retrospective analysis of the prediction accuracy was conducted after obtaining the observed data. For CL_total_, only the IVIVE method without ESF could predict human CL_total_ within twofold. This finding was consistent with the previous report that the IVIVE method using human microsomal data provided better prediction than the other methods for compounds eliminated primarily by CYP‐mediated mechanisms (Hosea et al., 2009). Two species allometry for Vd_ss_ without a protein binding correction showed a large discrepancy (13‐fold overprediction) between predicted and observed human oral CL_total_. Although the predicted human CL_total_ by the IVIVE method with ESF was comparable to the value predicted by FCIM, both methods overpredicted human oral CL_total_ by approximately fourfold. These results suggest that the similarity of predicted values by different prediction methods does not always indicate higher reliability. Even though FCIM was reported to yield the best predictions among various methods using larger data sets (Lombardo et al., 2013b), it failed to predict human CL_total_ of THY1773, which was possibly due to the lack of monkey data. The observed CL_renal_ was lower than the predicted value, suggesting the potential of renal reabsorption of THY1773 in humans, and indicating that the overprediction of human CL_total_ by IVIVE method with ESF was due to the overprediction of CL_liver_. The calculated ESF was not comparable across species, and similar results were obtained when the CL_int_ in hepatocytes were used for IVIVE (data not shown). The reasons for the discrepancy between in vitro and in vivo CL_int_ and species difference of ESF are not clear. These results indicate, however, the limitations of extrapolation of animal data to humans; that is, the unknown scaling factor obtained in animals should not be applied to human prediction without a reasonable hypothesis, especially when the value is not comparable across species. Recently, it has been reported that the IVIVE method using liver microsomes is highly predictive for CYP3A4 substrates than for other metabolic enzymes, and that the IVIVE method tends to underpredict CL_liver_ but the underprediction is CL_int_‐ and CL_liver_‐dependent (Bowman & Benet, 2019a; Hallifax, Foster, & Houston, 2010). Taken together with these reports, our results suggested that the IVIVE method would be the best method for the prediction of human CL_liver_ of a compound that is assumed to be metabolized primarily by CYP3A4 with low CL_int_.

Based on the retrospective analysis of the prediction accuracy of Vd_ss_, the rat–dog–human proportionality equation, a method without plasma protein binding correction, showed discrepancy (approximately threefold overprediction) between predicted and observed human oral Vd_ss_. The Øie‐Tozer method and rat single species scaling with Equation (5), both of which consider plasma protein binding, predicted human Vd_ss_ accurately (within twofold). Our results support the previous report that concluded the semi‐mechanistic Øie‐Tozer method as the most accurate method verified with more compounds (Lombardo et al., 2013a; Petersson, Papasouliotis, Lecomte, Badolo, & Dolgos, 2020).

The PBPK model using human liver microsomal CL_int_ without ESF (Model 1) described well the human plasma concentration–time profiles (Figure 4). In the PBPK model, as it was reported that CL_int_ in human intestinal microsomes and HLMs were not significantly different after normalization for tissue‐specific CYP3A abundance (Gertz, Harrison, Houston, & Galetin, 2010), CYP3A4 metabolism in the gut was estimated from CYP3A4 CL_int_ in HLMs, accounting for the differences in enzyme abundance between the liver and intestine. The fraction escaping gut first‐pass metabolism was predicted to be 0.97, and the bioavailability was predicted to be 95% by Model 1, which was almost consistent with our assumption for the evaluation of prediction accuracy of PK parameters.

Retrospective analysis of the PBPK/RO model also revealed the limitation of extrapolation of animal data to humans. According to the results of the phase 1 RO study using PET, the in vivo EC_50_ value for V_1B_ RO was comparable (within twofold) to the in vitro K_i_ value for human V_1B_ receptor binding inhibition (unpublished data). Based on the results of a rat V_1B_ RO study using a tritium labeled V_1B_ receptor ligand (Kamiya et al., 2020), the EC_50_ value for V_1B_ RO of THY1773 in the rat anterior pituitary was estimated to be 4.8 nmol/L, which was much lower than the in vitro K_i_ value of 80.1 nmol/L for rat anterior pituitary membrane V_1B_ receptor (unpublished data). This discrepancy (17‐fold higher affinity in vivo) was not likely to be explained by the target concentration because, even if the observed concentration ratio of the pituitary gland to plasma (4.7) was assumed to be applicable for free concentration, it would be much lower than the observed discrepancy. Also, hysteresis (time delay between target concentration and RO) was not likely to explain the discrepancy because hysteresis would result in a higher in vivo EC_50_ value than the in vitro K_i_ value, as the RO was evaluated at approximately t_max_. The discrepancy was possibly due to a more complex biological mechanism in vivo, but such a complex mechanism was difficult to include in the RO model and translate to humans based on the limited data obtained in the preclinical stage. For the prediction of a human effective dose, the discrepancy between in vitro K_i_ and in vivo EC_50_ observed in rats was not considered because there was no reasonable hypothesis that the discrepancy would be the same in humans, and we aimed to avoid the risk of lowering the probability of success of clinical trials by underpredicting a clinically effective dose. In addition, in the PET imaging study in rhesus monkeys, as previously reported (Koga et al., 2017), the monkey in vivo EC_50_ value of THY1773 for V_1B_ RO was similar to the in vitro K_i_ value for human recombinant V_1B_ receptor binding inhibition (unpublished data). Even though the in vitro K_i_ value for monkey V_1B_ receptor was not evaluated, we assumed that the monkey in vivo EC_50_ value for V_1B_ RO was comparable to the in vitro K_i_ value based on the high degree of V_1B_ receptor protein homology (96%) between human and monkey, according to the HomoloGene database (https://www.ncbi.nlm.nih.gov/homologene/22678). The aforementioned results provided the rationale for our approach. Retrospective analysis revealed that the discrepancy observed in rats was not observed in humans, and that the unknown scaling factor obtained in preclinical animal studies should not be applied to humans unless the mechanism is clear.

In summary, we established a human PBPK/RO model of THY1773, and succeeded in nearly accurately predicting a clinically effective dose for the treatment of MDD in the preclinical stage. To predict human PK parameters and a clinically effective dose more accurately in the preclinical stage, it is important to identify the most promising prediction method of various available methods. From our studies, it is suggested that mechanism based methods should be selected with reasonable assumptions for the successful predictions. Our studies also showed the limitations of extrapolation of animal data to humans. Caution should be exercised when applying an in vitro–in vivo scaling factor obtained in the preclinical animal studies to humans, especially when the reason for the in vitro–in vivo discrepancy is not clear.

## CONFLICT OF INTEREST

The authors declare no conflict of interest.
